# Potential Explanatory Factors for Higher Incident Hip Fracture Risk in Older Diabetic Adults

**DOI:** 10.1155/2011/979270

**Published:** 2011-08-07

**Authors:** Elsa S. Strotmeyer, Aruna Kamineni, Jane A. Cauley, John A. Robbins, Linda F. Fried, David S. Siscovick, Tamara B. Harris, Anne B. Newman

**Affiliations:** ^1^Department of Epidemiology, Center for Aging and Population Health, University of Pittsburgh, 130 North Bellefield Avenue, Room 515, Pittsburgh, PA 15213, USA; ^2^Group Health Research Institute, Seattle, WA 98101-2900, USA; ^3^Department of Medicine, University of California, Davis, CA 95817, USA; ^4^VA Pittsburgh Healthcare System and Renal-Electrolyte Division, School of Medicine, University of Pittsburgh, Pittsburgh, PA 15240, USA; ^5^The Cardiovascular Health Research Unit, Departments of Medicine and Epidemiology, University of Washington, Seattle, WA 98101, USA; ^6^Laboratory of Epidemiology, Demography and Biometry, Intramural Research Program, National Institute on Aging, Bethesda, MD 20892, USA; ^7^Division of Geriatric Medicine, School of Medicine, University of Pittsburgh, Pittsburgh, PA 15213, USA

## Abstract

Type 2 diabetes is associated with higher fracture risk. Diabetes-related conditions may account for this risk. Cardiovascular Health Study participants (*N* = 5641; 42.0% men; 15.5% black; 72.8±5.6 years) were followed 10.9 ± 4.6 years. Diabetes was defined as hypoglycemic medication use or fasting glucose (FG) ≥126 mg/dL. Peripheral artery disease (PAD) was defined as ankle-arm index <0.9. Incident hip fractures were from medical records. Crude hip fracture rates (/1000 person-years) were higher for diabetic vs. non-diabetic participants with BMI <25 (13.6, 95% CI: 8.9–20.2 versus 11.4, 95% CI: 10.1–12.9) and BMI ≥25 to <30 (8.3, 95% CI: 5.7–11.9 versus 6.6, 95% CI: 5.6–7.7), but similar for BMI ≥30. Adjusting for BMI, sex, race, and age, diabetes was related to fractures (HR = 1.34; 95% CI: 1.01–1.78). PAD (HR = 1.25 (95% CI: 0.92–1.57)) and longer walk time (HR = 1.07 (95% CI: 1.04–1.10)) modified the fracture risk in diabetes (HR = 1.17 (95% CI: 0.87–1.57)). Diabetes was associated with higher hip fracture risk after adjusting for BMI though this association was modified by diabetes-related conditions.

## 1. Introduction

Type 2 diabetic adults have an approximately 40–70% increased fracture risk [1, 2] compared to non-diabetic adults although the mechanism for this is not well established. Recent studies found fracture risk is elevated not just in the older diabetic adults, but also for middle-aged type 2 diabetic adults [3–6]. The risk appears to be equivalent for both diabetic men and women [7], suggesting an increased risk in both sexes. Importantly, in older diabetic adults, their generally higher bone mineral density (BMD) does not protect them from fracture and they have a higher fracture rate at an equivalent BMD to non-diabetic adults, which may potentially be due to their burden of diabetic complications [8, 9]. Although the higher weight of many type 2 diabetic patients is likely the main contributor to of their overall higher BMD [10], at an equivalent body size to a non-diabetic older adult, type 2 diabetic patients are more likely to fracture [9]. Therefore, other factors in type 2 diabetes besides BMD and obesity are likely contributing to the higher fracture rates. Identifying factors contributing to the higher fracture rate in diabetes may ultimately lead to preventative efforts for fracture in this high-risk population. 

Hyperglycemia itself may not directly account for the increased fracture risk in diabetes [1, 2] and impaired fasting glucose (IFG), or prediabetes, was not associated with higher fracture risk in older adults [9]. Emerging evidence suggests that clinical and subclinical alterations in peripheral nerve function [11], vascular function [12], and kidney function [13–15] are related to lower BMD, bone loss, or fracture in a dose-response manner. These complications could contribute to the higher fracture risk in diabetes. The objectives of the current study are to determine if type 2 diabetes or IFG are independently associated with a higher risk for hip fractures for older white and black men and women and if diabetes-related conditions contribute to the risk of hip fracture in older type 2 diabetic adults. 

## 2. Subjects, Materials, and Methods

### 2.1. Study Population

The CHS is a prospective, multicenter, cohort study of risk factors for cardiovascular disease in older community-dwelling adults. The study methods were previously described in detail [16]. In 1989-1990 (white cohort) and in 1992-1993 (black cohort), a total of 5888 noninstitutionalized, ambulatory men and women 65 years or older were enrolled from Medicare eligibility lists in four US communities (Pittsburgh, PA; Hagerstown, MD; Sacramento, CA; Winston-Salem, NC). Each center's institutional review committee approved the study and all participants gave informed consent prior to exams. Mean age at enrollment was 73 years (range: 65–100); 58% were women and 16% were black. Participants underwent an extensive baseline evaluation, including standardized clinical examinations, laboratory assessments, physical and cognitive functioning assessments, and questionnaires on medical history, health status, and risk factors, components of which were repeated at annual clinic visits through 1998/99. In 2005/06, the entire surviving CHS cohort was re-recruited to reevaluate physical and cognitive functioning for the CHS All Stars examination, an ancillary study to reassess functional status (median age 85, range 77–102; 66.5% female; 16.6% black) [17]. Phone followup for health outcomes was conducted every six months throughout the study and through the present time. Participants with hip fracture concurrent to motor vehicle accidents (*N* = 9) or pathologic fracture (*N* = 9), missing fasting glucose (*N* = 64), missing or noncompliant fasting status (*N* = 159), or missing information on hypoglycemic medications (*N* = 6) were excluded, resulting in 5,641 participants available for analyses. 

### 2.2. Hip Fractures

Information regarding hospitalizations was collected every 6 months from participants. To ensure completeness and verify the accuracy of the self-reported data, Medicare claims data were also used to identify any hospitalizations. Incident hip fractures were ascertained from a comprehensive review of hospitalization records through June 30, 2005, using International Classification of Diseases, Ninth Revision codes (ICD-9 codes 820.xx). Only the first hospitalization for hip fracture was considered and fractures were excluded if they were the result of excessive trauma (e.g., motor vehicle accident, ICD-9 E810-E825) or a pathologic condition (e.g., cancer, ICD-9 733.1). Among 5641 participants, 541 incident hip fractures were identified over 10.9 ± 4.6 years of followup. 

### 2.3. Diabetes

Baseline and incident diabetes status were defined based on medication information collected annually by medications inventories through 1998-1999, and fasting glucose (≥8 hours) measured on blood samples drawn in 1989-90, 1992-93, and 1996-97. Diabetes at study entry was defined as hypoglycemic medication use or a fasting glucose ≥126 mg/dL (≥7.0 mmol/L). Impaired fasting glucose (IFG) at study entry was defined as ≥100 mg/dL (≥6.1 mmol/L) but <126 mg/dL (<7.0 mmol/L) and no use of insulin or oral hypoglycemic medications. Participants were considered to have incident diabetes if either of the following conditions were met during the period after enrollment through a 1998-99 assessment: (1) any use of insulin or oral hypoglycemic medications or (2) fasting glucose ≥126 mg/dL. Detailed methods regarding blood draw, sample storage, quality assurance, and assay performance were previously described [18]. At baseline, of the 918 participants with prevalent diabetes, 361 were taking oral hypoglycemic medications only, 128 were taking insulin only, 11 were on both insulin and oral hypoglycemic medications, 417 were not taking medications, and 1 was missing medication information. 

### 2.4. Body Composition and Physical Function

Body weight was measured using a calibrated balance beam scale and height was measured with a wall-mounted stadiometer. Body mass index (BMI) was calculated in kg/m^2^. Waist circumference (cm) was measured at the umbilicus. Physical function was evaluated by the time in seconds to walk 15 feet in a corridor from a standing start. Participants self-reported a history of frequent falls in the past year.

### 2.5. Ankle-Arm Index

Subclinical peripheral arterial disease (PAD) was defined as ankle-arm index <0.9 (AAI), measured as the ratio of the ankle systolic blood pressure to the arm systolic blood pressure, using a standard protocol [19]. After a five minute rest lying on an examination table, a standard arm blood pressure cuff was used to measure systolic blood pressure in the right arm and then each ankle. After palpating the brachial and posterior tibial arteries and applying ultrasound gel, a Doppler stethoscope and standard mercury manometer were used to measure systolic blood pressure in the right brachial artery and each posterior tibial artery in rapid succession. The lower value of either the left or right AAI was used to classify subclinical PAD. 

### 2.6. Additional Covariates

Serum creatinine was measured using the Kodak Ektachem 700 Analyzer (Eastman Kodak, Rochester, NY), a colorimetric method. Kidney disease was defined as creatinine ≥1.5 mg/dL in men and ≥1.3 mg/dL in women [20] or an estimated glomerular filtration rate (eGFR) of <60 mL/min/1.73 m^2^ [21]. Fasting serum insulin level was measured by solid-phase immunoassay (Diagnostic Products Corp, Los Angeles, CA). Health histories collected at baseline included self-reported weight at age 50 years, current smoking, alcohol consumption, vision problems (unable to see to drive, to watch TV, or to recognize someone across a room with or without glasses) and clinical cardiovascular (CVD) disease (myocardial infarction, angina, congestive heart failure, claudication, stroke, transient ischemic attack). From the medication inventory, oral estrogen use was obtained [22], and participants were asked if they took any calcium supplements one or more times per week. Physical activity was calculated in kcal/week from total self-reported activities, excluding household chores, from the Minnesota Leisure Time Activities questionnaire [23]. 

### 2.7. Statistical Analyses

Univariate differences between DM, IFG, and normoglycemic groups were evaluated by chi-square tests for categorical covariates, by *t*-tests for normal continuous covariates, and by Mann-Whitney nonparametric tests for nonnormal continuous covariates, in men and women separately. Crude hip fracture rates (/1000 person-years) were determined for normal FG, IFG, and DM groups and also stratified by BMI categories of <25, ≥25 to <30, and >30. Cox proportional hazards models were used to estimate the relative risk (hazard ratio) of hip fracture associated with glycemic status. The participants' entry time into the analysis corresponded to their study enrollment date with time-at-risk until the earliest of incident hip fracture or censoring at death, loss-to-followup, or the last day of adjudicated followup (June 30, 2005). Multivariable models were adjusted for age, sex, race, BMI, diabetes, subclinical PAD (AAI < 0.9), and other potential confounders and mediators listed in the “Additional Covariates” section above. Sex did not satisfy the proportionality assumption due to an interaction of time with sex and therefore models were internally stratified for sex. Covariates described above in the Methods were retained in the final model if they attenuated the risk estimate by 10% or more or had a *P*-value < 0.10. Covariates correlated at *r* ≥ 0.50 were not entered simultaneously, but one was entered separately and then the other separately at the same step of the model, for example, BMI and waist circumference (*r* = 0.81); BMI and weight at age 50 (*r* = 0.50). For example, BMI was entered in the final regression model instead of waist circumference given the high correlation and the stronger relationship of BMI to hip fracture. Other covariates were not highly correlated, for example, smoking and subclinical PAD (*r* = 0.09) and smoking and alcohol use (*r* = 0.05). Potential effect modification of race, sex, BMI, and subclinical PAD with both DM and IFG were evaluated through multiplicative interaction terms with likelihood ratio tests. Stratified analyses by sex were performed for certain models a priori. Osteoporosis medication including bisphosphonates, calcitonin, and raloxifene, and thiazolidinediones (TZDs) were not used at the 1989-1990 baseline in any participants. However, a sensitivity analyses was performed which excluded 236 participants that took these medications during followup. Because incident diabetes in the normoglycemic and impaired fasting glucose groups may have modified the risk estimates for the diabetic group, a separate sensitivity analysis was done by excluding incident diabetes cases from these two groups. A sensitivity analysis was also done by excluding participants with AAI > 1.4, since this may represent vascular calcification and vessel stiffness. Analyses were performed using Stata software, version 10.0 (Stata Corp., College Station, Texas). 

## 3. Results

Participants with DM or IFG were more likely men (49.3% and 46.4% versus 36.4%; *P* < 0.001) than those with normal FG (Table 1). Participants with DM and IFG had a higher weight, a higher BMI, a greater self-reported weight at age 50 years, a higher waist circumference, a higher fasting insulin level, a lower eGFR (men only), lower physical activity, and were less likely to use oral estrogen or calcium supplements (women only) compared to those with normal FG. Additionally, participants with DM were more likely to be black, less likely to be a current drinker, had slower completion of the measured walk, and had more diabetes-related conditions (vision problems, subclinical PAD, prevalent CVD, and renal insufficiency) than those with normal FG. 

**Figure 1:**
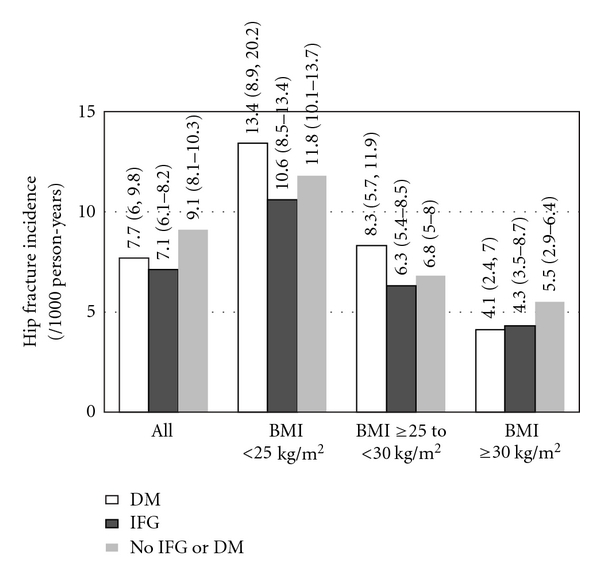
Crude incident hip fracture rate (/1000 person-years) by BMI category for diabetes mellitus, IFG, and normal FG.

Crude hip fracture rates (/1000 person-years) were 9.1 (95% CI: 8.1–10.3) for normal FG, 7.1 (95% CI: 6.1–8.2) for IFG, and 7.7 (95% CI: 6.0–9.8) for DM. Crude hip fracture rates were higher for diabetic compared to non-diabetic participants with BMI <25 (13.4, 95% CI: 8.9–20.2 versus 10.6, 95% CI: 8.5–13.4 IFG and 11.8, 95% CI: 10.1–13.7 normal FG) and BMI ≥25 to <30 (8.3, 95% CI: 5.7–11.9 versus 6.3, 95% CI: 5.0–8.0 IFG and 6.8, 95% CI: 5.4–8.5 normal FG), but were similar for BMI ≥30 (4.1, 95% CI: 2.4–7.0 versus 4.3, 95% CI: 2.9–6.4 IFG and 5.5, 95% CI: 3.5–8.7 normal FG) (Figure 1). The percentage of diabetic participants with fractures was 10.6% (23/218) for BMI <25, 7.5% (29/385) for BMI ≥25 to <30, and 4.2% (13/313) for BMI ≥30. The percentage of non-diabetic participants with fractures was 12.4% (243/1964) for BMI <25, 7.6% (149/1958) for BMI ≥25 to <30, and 5.6% (44/788) for BMI ≥30.

Diabetes was not significantly related to fractures in unadjusted models or models adjusted for sex, race, and age. The addition of BMI to the models adjusted for sex, race, and age increased the risk of hip fracture in DM participants (HR = 1.34; 95% CI: 1.01–1.78; Table 2). This indicated that the DM participants were at higher incidence fracture risk at an equivalent BMI to participants with normal glycemia. IFG was slightly protective for fracture in models minimally adjusted for age, sex, and race though this association was eliminated after adjusting for BMI and IFG was not shown to increase the risk of hip fracture (HR = 0.91; 95% CI: 0.75–1.11) in fully adjusted models. These results may indicate that higher BMI overall in the IFG group is protective for fracture but once BMI is adjusted for in the models, fracture risk is similar at an equivalent BMI to participants with normal glycemia. Excluding participants with incident diabetes (*N* = 103 from the normoglycemic group and *N* = 327 from the IFG group), did not appreciably change these estimates for DM (HR = 1.32; 95% CI: 1.00–1.76) or IFG (HR = 0.93; 95% CI: 0.76–1.15). Estimates were similar for diabetic women (HR = 1.35; 95% CI: 0.96–1.91) and men (HR = 1.31; 95% CI: 0.80–2.13), though lower statistical power likely made the results nonsignificant for these stratified analyses. IFG was not shown to increase the risk of hip fracture in separate analyses for women (HR = 0.94; 95% CI: 0.74–1.18) or men (HR = 0.86; 95% CI: 0.59–1.26). There were no interactions of race, sex, BMI, or subclinical PAD with glycemic status.

Addition of subclinical PAD (ankle-arm index <0.9) to the model *reduced* the HR for DM (1.25 (0.94–1.68)), rendering the association nonsignificant, and low AAI was significantly related to hip fracture (1.31 (1.01–1.71)). Excluding participants with AAI >1.4 (*N* = 67) did not change these results. Additional adjustment for current smoking, current drinking and 15 ft walk time further decreased the HR for DM (1.17 (0.87–1.58)), though each to a lesser degree than PAD, and decreased the HR for subclinical PAD (1.20 (0.92–1.57)) (Table 3). A longer time to complete the measured walk was significantly related to hip fracture incidence (1.07 (1.04–1.10)) and further reduced the HR for DM after the addition of current smoking and drinking to the models. Results did not change after excluding 236 participants taking osteoporosis medication during the followup period, including bisphosphonates, calcitonin, raloxifene, and thiazolidinediones (TZDs). 

## 4. Discussion

Our results indicate that diabetic participants with similar BMI as non-diabetic participants were more likely to fracture, particularly in the normal and overweight BMI categories. DM was associated with 34% higher hip fracture risk after adjusting for higher BMI in diabetic participants, consistent with previous estimates [1, 2]. One possibility for this observation is that sicker participants are losing weight due to advanced illnesses. Higher BMD loss among older diabetic women from the Health ABC Study was partly due to their greater weight loss over time [24]. The higher risk of fracture in the diabetic participants was partially modified by adding subclinical PAD to the models, which accounted for *a reduction in the *risk of fracture and made the primary association nonsignificant. This finding is important because although there is much evidence for the higher risk of fracture with DM [1–9, 25, 26], less is known about the factors that underlie this increased risk. Nearly all PAD in the elderly is subclinical, with 98% asymptomatic [19]. Although subclinical PAD is underappreciated clinically [27], it is preventable [28]. Although our overall attenuation with subclinical PAD was modest, our results, taken with previous findings of clinical and subclinical vascular disease associated with osteoporosis [12, 29–32], suggest that subclinical PAD may be a contributor to fractures in older diabetic populations. In the Health ABC Study the higher risk of fracture in diabetes persisted after adjustment for subclinical PAD [9], though this population was likely healthier at baseline than participants in our study. The association of vascular disease and osteoporosis may be the result of shared risk factors for which we adjusted for in the analyses (e.g., age, smoking) or shared biologic pathways (e.g., inflammatory cytokines, endogenous sex hormones) [33]. Alternatively, vascular disease may reduce blood flow to the lower extremities, including the hip, and modify bone metabolism to lead to osteoporosis.

Additional possible explanations for higher fracture risk in older adults with diabetes may be disease complications due to a long disease duration [1, 2, 34, 35], and/or specific complications such as impaired vision [25, 35] or neurologic impairments (e.g., neuropathy, stroke) [9, 25, 26, 36]. Further support exists from previous type 1 diabetes studies, summarized by Strotmeyer et al. [37], which indicate that lower BMD is associated with type 1 diabetic complications of nephropathy, neuropathy, and retinopathy. One reason for our lack of association between IFG and hip fracture may be that it is not the higher glucose levels per se that increase fracture risk but rather the diabetes-related conditions. Previous studies, including several meta-analyses, have also failed to find an association of IFG and fracture [1, 2, 9]. Falling and impaired motor abilities are more common in diabetic adults [9, 25, 26, 38, 39], and physical performance accounted for some of the increased risk for hip fractures in several cohorts of older adults [40, 41]. Our results indicated a slight modification of the higher fracture rate in DM once accounting for the slower walk time in DM participants. 

Our study was a large established cohort of black and white men and women followed for over a decade with an excellent ascertainment of hip fracture. However, we did not have a measure of BMD. Participants with higher BMI likely had a higher BMD as well, given the strong correlation usually observed between these two measures. A similar cohort which adjusted for the generally higher BMD observed in older diabetic adults found that diabetic participants with similar BMD as non-diabetic participants were more likely to be at risk for incident fractures and adding BMD to the models actually increased the strength of the association of fracture with DM [9]. We did not have measures of diabetes duration or of peripheral nerve function. Poor peripheral nerve function may increase the risk for falls [42] and has been related to lower bone mineral density [11, 37]. Additionally, we did not assess vitamin D deficiency, which is associated with osteoporosis and has been linked to diabetes and vascular disease [43]. TZDs, although associated with fracture in diabetic women [44], were introduced late in our study followup period and our results were unaffected when we excluded participants using TZDs during the followup period for the fracture outcome. Furthermore, the use of these medications were likely not prevalent enough to account for the relationship of DM and fracture and past studies indicating higher fracture risk in type 2 diabetic adults were conducted prior to the introduction and widespread use of TZD medication [45]. Although we were not able to fully address this issue, hypoglycemic medication use is important to evaluate in the context of diabetes and fracture and it is critical to recognize the potential for confounding by indication. Finally, older diabetic adults with more severe disease may have been less likely to participate in our study and this may have made our risk estimates for fracture more conservative.

Likely diabetes-related conditions account for a portion of increased fracture risk and potentially BMD changes; however, these have not been comprehensively evaluated. Our results indicated that subclinical PAD and slower walking time be related to a part of the higher risk for hip fracture in older diabetic adults. PAD is preventable and treatable [27, 28] and clinical PAD was recently linked to a three-time higher risk of hip fracture [46]. Other diabetes-related complications may be important as well and should be investigated for their role in fractures in older diabetic adults. Poor physical function in older diabetic adults could also be improved through physical therapy or exercise interventions. Potentially, preventing diabetes-related conditions in older diabetic adults may reduce fractures in this population. 

##  Conflict of Interests

The authors report no conflicts of interest. 

## Figures and Tables

**Table 1:** Baseline descriptive characteristics by glycemic status for 5,641 women and men in the CHS.

	Women (*n* = 3254)			Men (*n* = 2387)		
	DM (*n* = 465)	IFG (*n* = 1144)	No IFG or DM (*n* = 1645)	DM (*n* = 453)	IFG (*n* = 991)	No IFG or DM (*n* = 943)
Black race	139 (29.9%)^†^	162 (14.2%)	248 (15.1%)	84 (18.5%)	100 (10.1%)^‡^	142 (15.1%)
Age (years)	72.7 (±5.7)	72.6 (±5.5)	72.4 (±5.3)	72.9 (±5.2)	73.2 (±5.6)	73.5 (±6.1)
Current smoker	50 (10.8%)	149 (13.0%)	213 (13.0%)	42 (9.3%)	105 (10.6%)	118 (12.5%)
Current drinker	122 (26.3%)^†^	535 (46.9%)	777 (47.4%)	189 (41.8%)^†^	623 (63.2%)	572 (60.9%)
Oral estrogen use	20 (4.3%)^†^	102 (8.9%)^†^	270 (16.4%)	NA	NA	NA
Calcium supplement use	73 (15.9%)^†^	273 (24.1%)^†^	513 (31.5%)	35 (7.8%)	95 (9.7%)	92 (9.9%)
Height (cm)	159.4 (±6.3)	158.9 (±6.4)	158.7 (±6.1)	173.6 (±6.8)	172.9 (±6.6)	173.0 (±6.5)
Weight (lbs)	165.2 (±32.3)^†^	154.4 (±31.2)^†^	141.6 (±28.3)	184.2 (±31.2)^†^	177.2 (±26.8)^†^	167.1 (±24.8)
BMI (kg/m^2^)	29.5 (±5.4)^†^	27.7 (±5.2)^†^	25.5 (±4.8)	27.7 (±4.3)^†^	26.9 (±3.7)^†^	25.3 (±3.3)
Waist circumference (cm)	100.4 (±14.1)^†^	94.1 (±14.1)^†^	88.4 (±13.5)	101.2 (±11.2)^†^	98.8 (±10.0)^†^	94.6 (±9.5)
Weight at age 50 (lbs)	154.7 (±28.7)^†^	141.2 (±23.1)^†^	135.3 (±22.1)	184.3 (±29.7)^†^	172.6 (±24.1)^†^	167.1 (±21.5)
Fasting insulin (IU/mL)	21 (14,31)^∗†^	14 (11,19)^∗†^	11 (8,14)*	17 (12,28)^∗†^	14 (10,19)^∗†^	11 (8,14)*
Walk time (s to walk 15 ft)	6.7 (±2.7)^†^	5.9 (±2.1)	5.9 (±2.3)	6.0 (±3.1)^‡^	5.5 (±2.0)	5.5 (±2.2)
Physical activity (kcal/wk)*	210 (0,749)^∗†^	385 (28,1094)^∗‡^	495 (79,1193)*	856 (230,1920)^∗‡^	917 (306,2166)^∗‡^	1088 (405,2407)*
Frequent falls	33 (7.1%)^†^	38 (3.3%)	72 (4.4%)	16 (3.6%)	13 (1.3%)	23 (2.5%)
Vision problem	53 (12.4%)^‡^	83 (7.7%)	117 (7.6%)	18 (4.0%)	50 (5.1%)	55 (6.0%)
AAI < 0.90	89 (20.1%)^†^	123 (11.1%)	180 (11.2%)	102 (23.2%)^†^	129 (13.2%)	112 (12.0%)
Prevalent CVD	151 (32.5%)^†^	237 (20.7%)^‡^	285 (17.3%)	189 (41.7%)^†^	285 (28.8%)	290 (30.8%)
High creatinine (≥1.5 mg/dL men or ≥1.3 mg/dL women)	37 (8.2%)^‡^	52 (4.6%)	70 (4.3%)	55 (12.2%)	108 (10.9%)	95 (10.1%)
eGFR <60 mL/min/ 1.73 m^2^	99 (21.9%)	245 (21.4%)	333 (20.2%)	118 (26.5%)^‡^	245 (24.7%)^‡^	196 (20.8%)

Data are means (±standard deviations) or proportions unless otherwise indicated.

*Data are medians (25th percentile, 75th percentile).

^†^ *P* value <0.001. ^‡^ *P* value <0.05.

**Table 2:** Association of impaired fasting glucose and diabetes mellitus with incident hip fracture.

	No IFG or DM	IFG	Diabetes
Overall (*n*)	2588	2135	918
No. of hip fractures	269	169	65
Person-years	29,462	23,851	8,428

	Multivariable HR (95% CI)		

Model 1: Age-sex-race adjusted	1.0 (ref.)	0.79 (0.65–0.96)	1.05 (0.80–1.39)
Model 2: Model 1 + BMI	1.0 (ref.)	0.91 (0.75–1.11)	1.34 (1.01–1.77)
Model 3: Model 2 + AAI < 0.9	1.0 (ref.)	0.92 (0.75–1.12)	1.25 (0.93–1.67)

**Table 3:** Final model for association of impaired fasting glucose and Diabetes Mellitus with incident hip fracture.*

	HR	95% CI
DM	1.17	0.87–1.57
IFG	0.93	0.76–1.13
AAI, <0.9	1.20	0.92–1.57
BMI, kg/m^2^	0.93	0.91–0.95
Time for walk, s	1.07	1.04–1.10

*Models were internally stratified for sex and adjusted for age, race, current smoking, and current alcohol use. Clinical cardiovascular disease, use of oral estrogen, use of calcium supplements, renal insufficiency (either creatinine ≥1.5 mg/dL in men/≥1.3 mg/dL in women or eGFR <60 mL/min/1.73 m^2^), fasting insulin level, physical activity, history of falls, and vision problems were not included in the final model since they did not attenuate the HR for diabetes and were not significantly related to hip fracture.
